# Cadmium Induces Kidney Iron Deficiency and Chronic Kidney Injury by Interfering with the Iron Metabolism in Rats

**DOI:** 10.3390/ijms25020763

**Published:** 2024-01-07

**Authors:** Kanglei Zhang, Mengfei Long, Wenxuan Dong, Jiahui Li, Xueru Wang, Wenjing Liu, Qing Huang, Yuyu Ping, Hui Zou, Ruilong Song, Gang Liu, Di Ran, Zongping Liu

**Affiliations:** 1 College of Veterinary Medicine, Yangzhou University, 12 East Wenhui Road, Yangzhou 225009, China; zhangkl93@163.com (K.Z.); mflone@163.com (M.L.); dongwenxuan7@163.com (W.D.); ljhmiuky@163.com (J.L.); xueruwang9069@163.com (X.W.); liuwenjing00000@163.com (W.L.); h15190894489@163.com (Q.H.); 13722107278@163.com (Y.P.); zouhui@yzu.edu.cn (H.Z.); srlbio@163.com (R.S.); gangliu@yzu.edu.cn (G.L.); 2 Jiangsu Co-Innovation Center for Prevention and Control of Important Animal Infectious Diseases and Zoonoses, Yangzhou 225009, China; 3 Joint International Research Laboratory of Agriculture and Agri-Product Safety, the Ministry of Education of China, Yangzhou University, Yangzhou 225009, China; 4 College of Medicine, Tulane University, New Orleans, LA 70112, USA; 5 College of Veterinary Medicine, Southwest University, Chongqing 400715, China; diran@uic.edu; 6 College of Medicine, University of Illinois at Chicago, Chicago, IL 60607, USA

**Keywords:** cadmium, kidney, proximal tubular cells, iron metabolism, ferroptosis

## Abstract

Cadmium (Cd) is a common environmental pollutant and occupational toxicant that seriously affects various mammalian organs, especially the kidney. Iron ion is an essential trace element in the body, and the disorder of iron metabolism is involved in the development of multiple pathological processes. An iron overload can induce a new type of cell death, defined as ferroptosis. However, whether iron metabolism is abnormal in Cd-induced nephrotoxicity and the role of ferroptosis in Cd-induced nephrotoxicity need to be further elucidated. Sprague Dawley male rats were randomly assigned into three groups: a control group, a 50 mg/L CdCl2-treated group, and a 75 mg/L CdCl2-treated group by drinking water for 1 month and 6 months, respectively. The results showed that Cd could induce renal histopathological abnormalities and dysfunction, disrupt the mitochondria’s ultrastructure, and increase the ROS and MDA content. Next, Cd exposure caused GSH/GPX4 axis blockade, increased FTH1 and COX2 expression, decreased ACSL4 expression, and significantly decreased the iron content in proximal tubular cells or kidney tissues. Further study showed that the expression of iron absorption-related genes *SLC11A2*, *CUBN*, *LRP2*, *SLC39A14*, and *SLC39A8* decreased in proximal tubular cells or kidneys after Cd exposure, while TFRC and iron export-related gene *SLC40A1* did not change significantly. Moreover, Cd exposure increased *SLC11A2* gene expression and decreased *SLC40A1* gene expression in the duodenum. Finally, NAC or Fer-1 partially alleviated Cd-induced proximal tubular cell damage, while DFO and Erastin further aggravated Cd-induced cell damage. In conclusion, our results indicated that Cd could cause iron deficiency and chronic kidney injury by interfering with the iron metabolism rather than typical ferroptosis. Our findings suggest that an abnormal iron metabolism may contribute to Cd-induced nephrotoxicity, providing a novel approach to preventing kidney disease in clinical practice.

## 1. Introduction

Cadmium (Cd), a common environmental heavy metal pollutant and occupational poison, has caused significant concern due to its serious threat to food safety and public health [1,2]. Moreover, with the continuous development of industrialization, Cd pollution to the environment is becoming more serious [3]. Cd can be absorbed into the body through the digestive tract, respiratory tract, and skin contact routes. Importantly, absorbed Cd is difficult to remove from the body and has a long biological half-life of 10–30 years [4,5]. Studies have found that Cd exposure damages various tissues and organs, closely related to developing acute or chronic diseases [6,7,8]. As the organ of excretion of internal metabolic waste, the kidney is one of the main targeted organs of Cd accumulation and toxicity. Long-term exposure to Cd can cause irreversible damage to the kidney [9,10,11]. Notably, the deterioration of proximal tubular cells is the main cause of Cd-induced kidney disease, associated with multiple mechanisms, including essential element replacement, oxidative stress, inflammatory response, autophagy, apoptosis, and epigenetic changes [12,13,14,15].

Systemic and cellular iron homeostasis strictly depends on normal iron metabolism, including iron import, utilization, storage, and export. Absorption of dietary iron, including both heme and nonheme forms, in the duodenum requires the co-regulation of a variety of proteins or transporters, such as heme carrier protein 1 (HCP1), duodenal cytochrome B (Dcytb), solute carrier family 11 member 2 (SLC11A2), solute carrier family 40 member 1 (SLC40A1), and ferroxidase hephaestin (Heph) [16]. Subsequently, the circulating iron is transferred to various organs with transferrin (TF) [17]. In the kidney, TF-bound iron is filtered by the glomerulus and reabsorbed by transferrin receptor 1 (TFRC) and Megalin–Cubulin complexes that are internalized at the tubular site. Non-TF-bound iron is mainly transported by the divalent metal transporters SLC11A2, solute carrier family 39 member 8 (SLC39A8), and solute carrier family 39 member 14 (SLC39A14) in the proximal and distal tubular cells [18,19]. Intracellular iron enters the “labile iron pool” for subsequent metabolic utilization or temporary storage. The iron and Cd metabolism in vivo interfere with each other. Iron deficiency has been found to lead to increased absorption and storage of Cd in the body [20]. Likewise, Cd can also cause abnormal iron metabolism, resulting in an overload or deficiency of iron in the body [21,22,23]. However, whether a disorder of iron metabolism is involved in Cd-induced chronic kidney injury needs to be further explored.

Ferroptosis, a regulatory cell death form driven by increased intracellular iron content and subsequent accumulation of lipid peroxidation, has been implicated in the development of many diseases, such as tumors, nervous system diseases, acute kidney injury, and ischemia/reperfusion [24,25,26]. Free radical production, fatty acid supply, and lipid peroxidation are critical for ferroptosis [27]. The underlying molecular mechanism of ferroptosis involves alterations in various related proteins such as solute carrier family 7 member 11 (SLC7A11), glutathione peroxidase 4 (GPX4), acyl-CoA synthetase long chain family member 4 (ACSL4), acyl-CoA synthetase family member 2 (ACSF2), iron response element binding protein 2 (IREB2), and ferritin heavy chain 1 (FTH1) [26,28]. Studies have shown that although Cd is not an oxidation-reducing metal, it can disrupt the homeostasis of the oxidation–antioxidant system and cause oxidative stress by replacing key prosthetic groups of antioxidant enzymes, reducing the activity of antioxidant enzymes and increasing the production of ROS [13,29]. In recent years, certain pioneering studies have demonstrated a crosstalk between metal toxicity and ferroptosis [30,31]. However, whether ferroptosis is involved in Cd-induced chronic kidney injury and the related mechanisms has yet to be fully elucidated.

Therefore, this study aims to investigate whether abnormal iron metabolism or ferroptosis is involved in Cd-induced nephrotoxicity using in vitro and in vivo models. Our findings will provide new insights into the mechanisms of chronic kidney injury induced by Cd exposure and may open new avenues for preventing kidney disease in clinical practice.

## 2. Results

### 2.1. Cd Induced Oxidative Stress and Chronic Kidney Injury in Rats In Vivo and In Vitro

**Figure 1:**
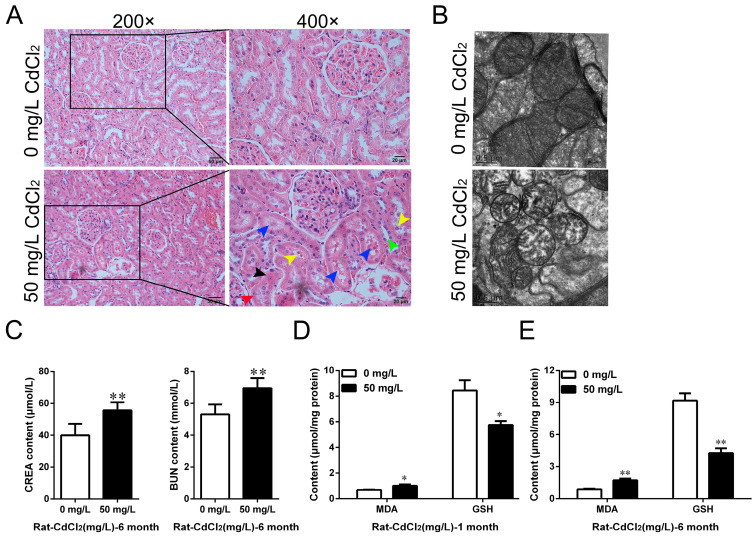
Effects of cadmium exposure on oxidative stress and kidney injury in rats. (**A**) Renal histopathological structures were observed by H&E staining and showed a loss of the tubular brush border (green arrow), swelling (black arrow), vacuolization (red arrow) or detachment (yellow arrow) of epithelial cells, and protein coagulation (blue arrow) in the tubular lumen after Cd treatment; scale = 50 μM or 20 μM. (**B**) Ultrastructure of mitochondria in rat kidney tissues was observed by transmission electron microscope (TEM); scale = 0.5 μM. (**C**) The levels of CREA and BUN in serum were detected using automatic biochemical analyzer. (**D**,**E**) MDA and GSH contents in kidney tissue were detected after 1 month and 6 months of Cd treatment. Each experiment was duplicated at least three times. (* *p* < 0.05, ** *p* < 0.01).

Male rats were treated by feeding water for 1 month and 6 months to study the toxicity of Cd in the kidney. H&E staining was performed to observe the histopathological changes in the kidney. The results showed that Cd exposure induced significant tubular morphological disruptions, mainly characterized by a loss of the tubular brush border (green arrow), swelling (black arrow), vacuolization (red arrow) or detachment (yellow arrow) of epithelial cells, and protein coagulation (blue arrow) in the tubular lumen (Figure 1A). The mitochondrial ultrastructure was observed under transmission electron microscopy. The results showed that Cd treatment decreased the mitochondrial matrix electron density, disrupted the integrity of the outer mitochondrial membrane, and caused the breakage and disappearance of mitochondrial cristae (Figure 1B). The results of serum biochemical assays showed that compared with the control group, the levels of creatinine (CREA) and blood urea nitrogen (BUN) increased by 30.92% (*p* < 0.01) and 39.37% (*p* < 0.01), respectively, after treatment with 50 mg/L Cd for 6 months (Figure 1C). Malondialdehyde (MDA) and glutathione (GSH) were detected in kidney tissues. The results showed that compared with the control group, the content of MDA increased by 48.48% (*p* < 0.05), and the content of GSH decreased by 31.84% (*p* < 0.05) after 50 mg/L Cd treatment for 1 month (Figure 1D). After 6 months of 50 mg/L Cd treatment, the content of MDA increased by 94.58% (*p* < 0.01), and the content of GSH decreased by 53.51% (*p* < 0.01) (Figure 1E).

**Figure 2:**
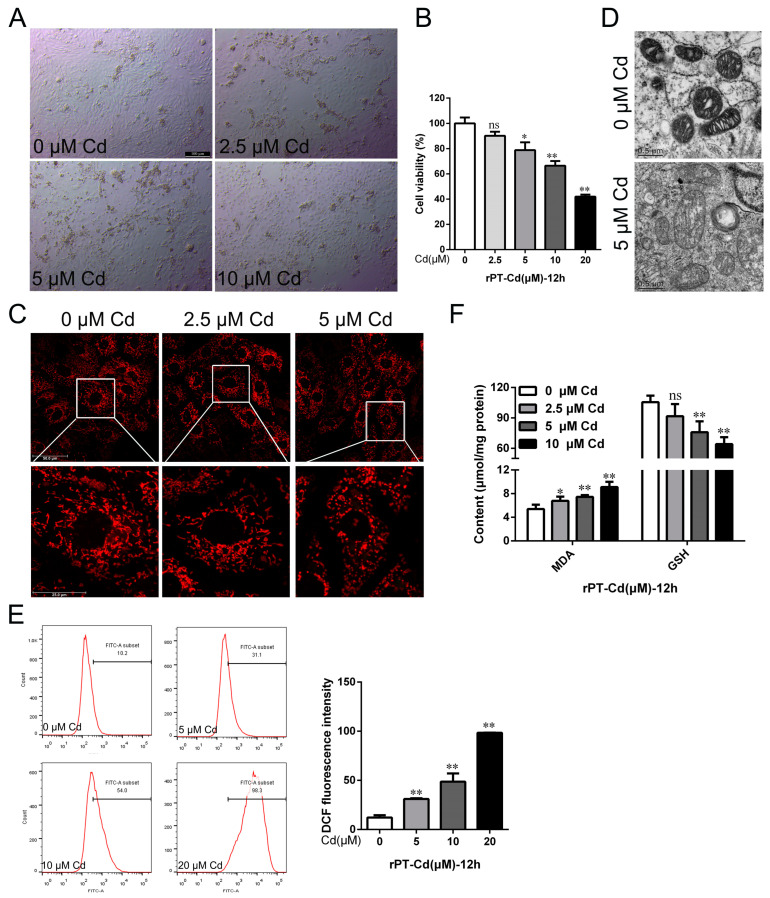
Effects of cadmium exposure on oxidative stress and cell injury in rPT cells. (**A**) Cell morphology was observed by phase-contrast microscopy; scale = 100 μM. (**B**) Cell viability was detected by CCK-8 assay. (**C**) The mitochondrial reticular structure was observed by confocal fluorescence microscopy after staining with Mito-Tracker Red probe; scale = 25 μM. (**D**) Mitochondrial ultrastructure in rPT cells was observed by transmission electron microscopy; scale = 0.5 μM. (**E**) Intracellular ROS levels were detected by flow cytometry after staining with DCFH-DA probe. (**F**) Intracellular MDA and GSH contents were measured using the corresponding assay kits. Each experiment was duplicated at least three times. (ns, not significant; * *p* < 0.05, ** *p* < 0.01).

The primary rat proximal tubular cells (rPT cells) were isolated and cultured to further elucidate the mechanism of Cd-induced nephrotoxicity in vitro. Morphological changes in rPT cells were observed under a phase-contrast microscope. The results showed that with the increase in Cd concentration, the cell density gradually decreased, the cell morphology was wrinkled and rounded or vacuolated, and some cells were detached (Figure 2A). The results of the CCK-8 assay showed that 5 μM Cd reduced the cell viability by 21.23% (*p* < 0.05), and the cell viability decreased with an increasing Cd concentration (Figure 2B). After staining with a Mito-Tracker Red probe, the reticular structure of mitochondria was observed under confocal fluorescence microscopy. The results showed that Cd exposure disrupted mitochondrial reticular structural integrity, showing fragmented, scattered distributions (Figure 2C). The Cd treatment significantly disrupted the ultrastructure of mitochondria in rPT cells (Figure 2D). Moreover, the intracellular reactive oxygen species (ROS) content, as well as the MDA and GSH contents, were examined to assess the oxidative stress and lipid peroxidation levels. The results showed that compared with the control group, the 5, 10, and 20 μM Cd-treated group increased intracellular ROS content by 154.37% (*p* < 0.01), 298.63% (*p* < 0.01), and 706.83% (*p* < 0.01), respectively (Figure 2E). Meanwhile, MDA content increased by 25.41% (*p* < 0.05), 37.85% (*p* < 0.01), and 68.77% (*p* < 0.01), and GSH content decreased by 13.16% (*p* > 0.05), 28.11% (*p* < 0.01), and 39.17% (*p* < 0.01) in the 2.5, 5, and 10 μM Cd groups, respectively (Figure 2F). The above results suggest that Cd exposure induced oxidative stress and chronic kidney injury in vivo and in vitro.

### 2.2. Cd Failed to Induce Typical Ferroptosis of Rat Kidneys In Vivo and In Vitro

**Figure 3:**
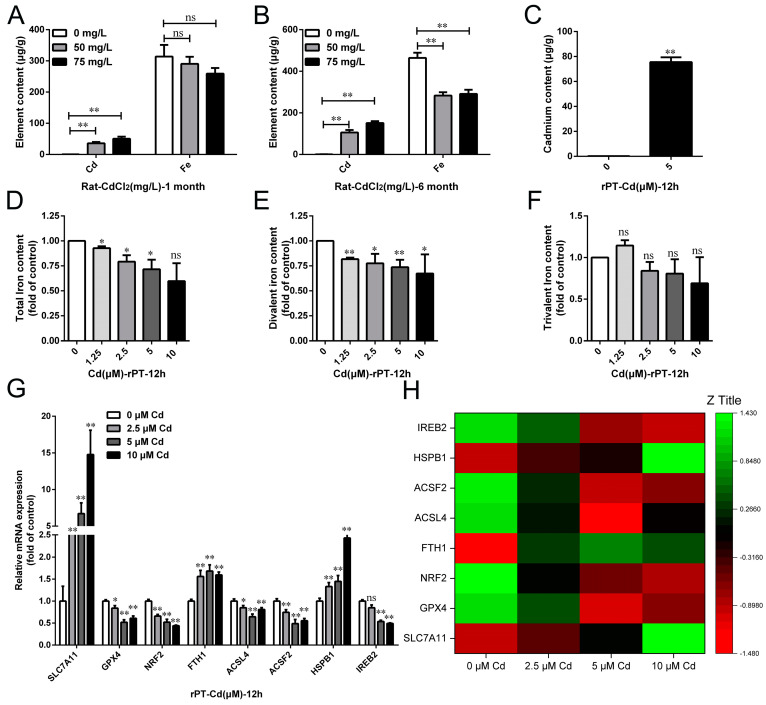
Effects of cadmium exposure on renal iron content and ferroptosis-related gene expression in rats in vivo and in vitro. Cd and iron contents were detected in kidney tissues after 1 month (**A**) and 6 months (**B**) of Cd exposure by flame atomic absorption spectrophotometer. (**C**) Intracellular Cd was detected by flame atomic absorption spectrophotometer. Intracellular total Fe (**D**), divalent Fe (**E**), and ferric Fe (**F**) contents were detected by iron ion detection kit after cells were treated with Cd for 12 h. (**G**) Expression levels of ferroptosis-related genes (*SLC7A11*, *GPX4*, *NRF2*, *FTH1*, *ACSL4*, *ACSF2*, *HSPB1*, and *IREB2*) were examined by qRT-PCR. (**H**) Heat map of ferroptosis-related gene expression was generated by Origene 2021 software. Each experiment was duplicated at least three times. (ns, not significant; * *p* < 0.05, ** *p* < 0.01).

We further investigated whether ferroptosis is involved in Cd-induced kidney injury. The Cd and Fe contents in dried kidney tissues were measured by flame atomic spectrophotometer. The results showed that after 1 month of 50 and 75 mg/L Cd treatment, the Cd content was 63.10-fold (*p* < 0.01) and 89.83-fold (*p* < 0.01) higher than that in the control group, respectively, while the Fe content decreased by 7.38% (*p* > 0.05) and 17.40% (*p* > 0.05), respectively (Figure 3A). After 6 months of 50 and 75 mg/L Cd treatment, the Cd content increased by 114.25-fold (*p* < 0.01) and 164.13-fold (*p* < 0.01) compared with the control group, respectively, while Fe content decreased by 38.98% (*p* < 0.01) and 37.34% (*p* < 0.01), respectively (Figure 3B). At the same time, the Cd and Fe contents in rPT cells were examined. The results showed that the Cd content in the 5 μM Cd-treated group was 348.70-fold (*p* < 0.01) higher than in the control group. The contents of total iron in the 1.25, 2.5, 5, and 10 μM Cd-treated group decreased by 7.22% (*p* < 0.01), 20.83% (*p* < 0.05), 28.35% (*p* < 0.05), and 40.21% (*p* > 0.05), and the contents of divalent iron decreased by 18.29% (*p* < 0.01), 22.49% (*p* < 0.01), 26.33% (*p* < 0.01), and 32.62% (*p* < 0.05), respectively (Figure 3D,E). However, there was no significant change in the content of ferric iron in each treatment group (*p* > 0.05) (Figure 3F).

**Figure 4:**
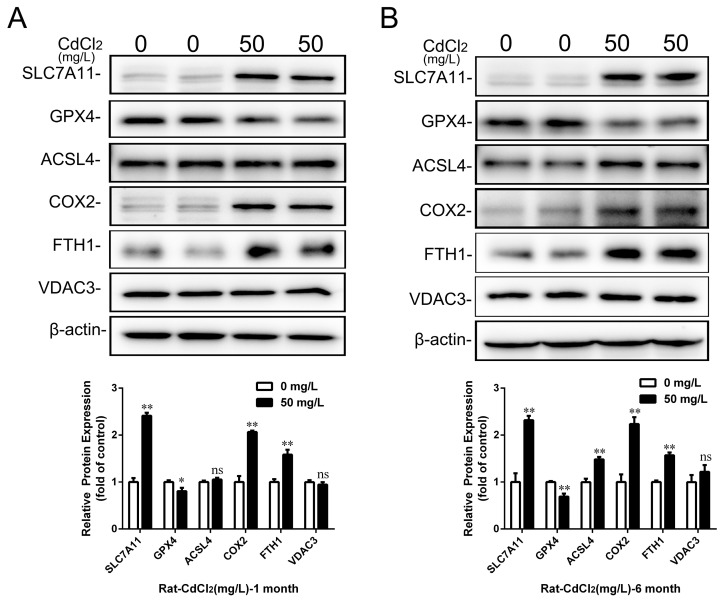
The effect of cadmium exposure on the expression of ferroptosis-related proteins in kidneys of rats in vivo. Representative Western blot images and quantitative analysis of ferroptosis-related proteins (SLC7A11, GPX4, ACSL4, COX2, FTH1, and VCAD3) in total cellular lysates after 1 month (**A**) and 6 months (**B**) of Cd exposure. Each experiment was duplicated at least three times. (ns, not significant; * *p* < 0.05, ** *p* < 0.01).

**Figure 5:**
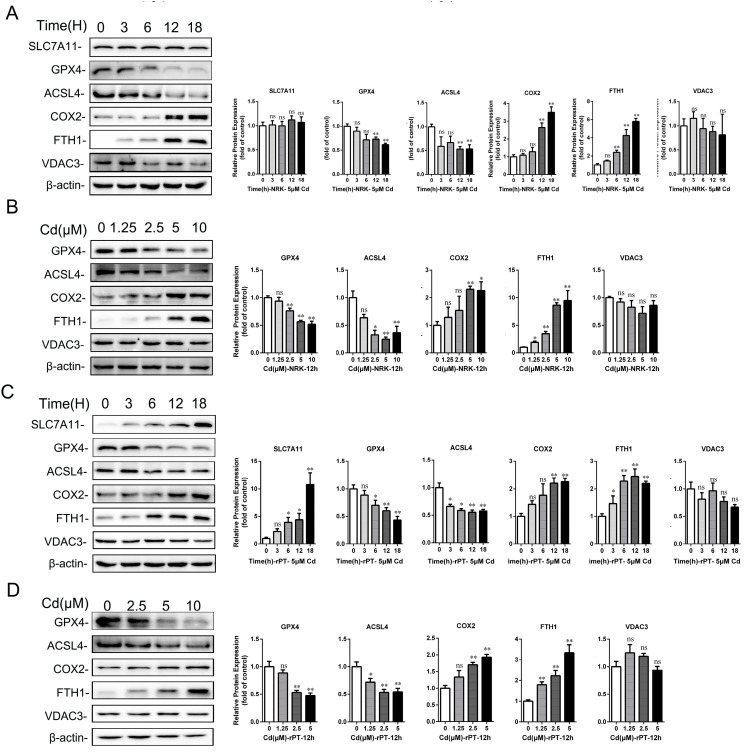
The effect of cadmium exposure on the expression of ferroptosis-related proteins in rPT or immortalized rat proximal tubule cells (NRK-52E cells). Western blot images and quantitative analysis of ferroptosis-related proteins (SLC7A11, GPX4, ACSL4, COX2, FTH1, and VCAD3) in total cellular lysates of NRK-52E or rPT cells after 2.5 or 5 μM Cd treatment for different lengths of time (**A**,**C**) and different concentrations of Cd for 12 h (**B**,**D**). Each experiment was duplicated at least three times. (ns, not significant; * *p* < 0.05, ** *p* < 0.01).

Next, ferroptosis-related gene and protein expression levels were analyzed by Western blot and qRT-PCR. The results showed that the gene expressions of *SLC7A11*, *HSPB1*, and *FTH1* were significantly elevated, while *GPX4*, *ACSF2*, *ACSL4*, *IREB2*, and *NRF2* were decreased considerably in rPT cells after Cd treatment (*p* < 0.05 or *p* < 0.01) (Figure 3G,H). The Western blot results showed that the protein expression of SLC7A11, COX2, and FTH1 increased by 141.29% (*p* < 0.01), 106.57% (*p* < 0.01), and 58.40% (*p* < 0.01), respectively, while GPX4 decreased by 19.27% (*p* < 0.05) after 50 mg/L Cd treatment for 1 month compared with the control group (Figure 4A). After 50 mg/L Cd treatment for 6 months, SLC7A11, COX2, FTH1, and ACSL4 protein expression increased by 131.76% (*p* < 0.01), 123.52% (*p* < 0.01), 57.12% (*p* < 0.01), and 48.47% (*p* < 0.01), respectively, while the GPX4 protein level decreased by 30.85% (*p* < 0.01) (Figure 4B). In addition, the results for ferroptosis-related protein expression in vitro showed that GPX4 and ACSL4 protein expression significantly decreased (*p* < 0.05 or *p* < 0.01), COX2, FTH1, or SLC7A11 protein expression increased (*p* < 0.05 or *p* < 0.01), while VDAC3 protein expression did not change significantly (*p* > 0.05) with the increase in Cd treatment time or concentration (Figure 5A–D).

### 2.3. Cd Reduced Circulating Iron Content by Reducing Iron Absorption in the Small Intestine

**Figure 6:**
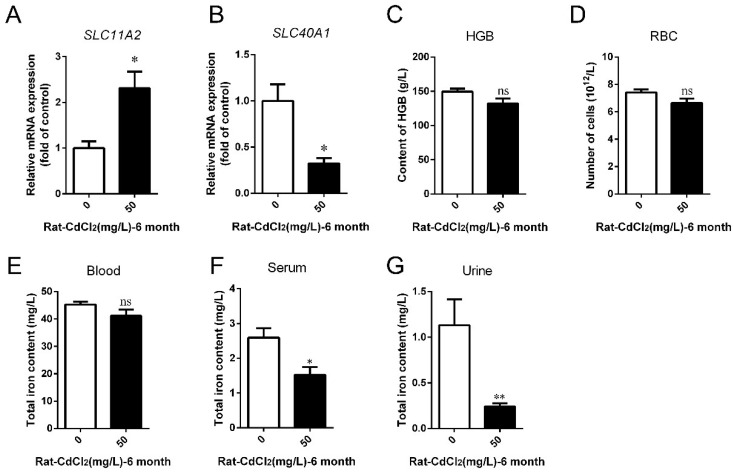
Effects of cadmium treatment on iron absorption and excretion in rats. After 6 months of Cd exposure, the expression levels of *SLC11A2* (**A**) and *SLC40A1* (**B**) genes in duodenal epithelial cells were detected by qRT-PCR. HGB content (**C**) and RBC count (**D**) in whole blood were detected by automatic blood analyzer. The iron content in whole blood (**E**), serum (**F**), and urine (**G**) was detected by flame atomic spectrophotometer. Each experiment was duplicated at least three times. (ns, not significant; * *p* < 0.05, ** *p* < 0.01).

The previous results showed that the iron content was reduced in kidney tissues or cells after Cd treatment, so we next explored the mechanism of iron deficiency caused by Cd exposure. The expression levels of iron transport-related genes in the small intestine were analyzed. The results showed that the transcript level of *SLC11A2* increased by 131.30% (*p* < 0.05), but the transcription level of *SLC40A1* decreased by 67.71% (*p* < 0.05) after 6 months of 50 mg/L Cd treatment (Figure 6A,B). In addition, we examined the hemoglobin (HGB) content and red blood cell (RBC) count in the blood and iron content in serum to reflect the body’s iron storage. The results showed that the content of HGB decreased by 11.87% (*p* > 0.05), and the RBC count decreased by 10.28% (*p* > 0.05) after 50 mg/L Cd treatment compared with the control group (Figure 6C,D). The Fe content in whole blood, serum, and urine decreased by 9.11% (*p* > 0.05), 41.15% (*p* < 0.05), and 78.80% (*p* < 0.05), respectively, in the 50 mg/L Cd-treated group (Figure 6E–G). These results suggested that Cd exposure caused a decrease in iron absorption in the duodenum and decreased circulating iron content, which might be related to iron deficiency in the kidney and proximal tubular cells of rats.

### 2.4. Cd Induced the Disorder of Iron Metabolism in Kidney and Proximal Tubular Cells of Rats

**Figure 7:**
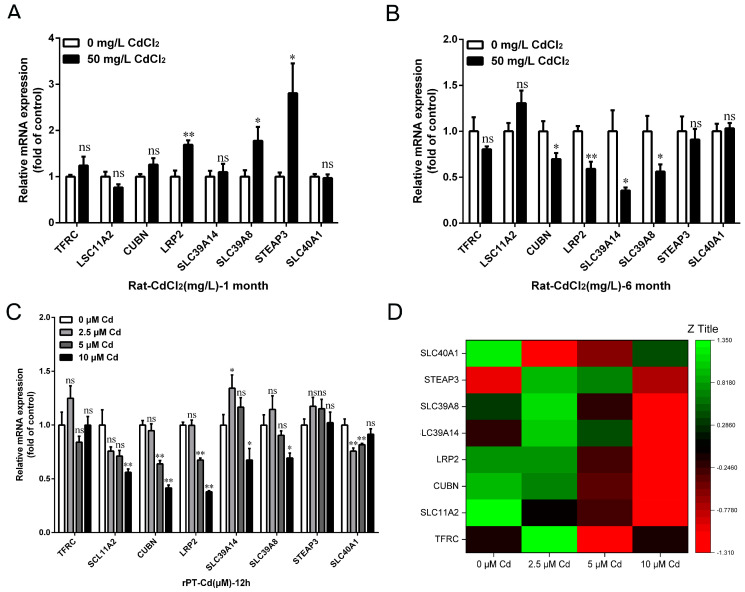
Effects of cadmium exposure on renal iron metabolism in rats in vivo and in vitro. Expression levels of iron metabolism-related genes (*TFRC*, *SLC11A2*, *CUBN*, *LRP2*, *SLC39A14*, *SLC39A8*, *STEAP3*, and *SLC40A1*) were examined in kidney after 1 month (**A**) and 6 months (**B**) of Cd exposure, and rPT cells were treated with different concentrations of Cd for 12 h (**C**) by qRT-PCR. (**D**) Heat map of iron metabolism-related gene expression after rPT cells were treated with different concentrations of Cd for 12 h, generated by Origene 2021 software. Each experiment was duplicated at least three times. (ns, not significant; * *p* < 0.05, ** *p* < 0.01).

We further investigated the effect of Cd on iron metabolism in kidneys and proximal tubular cells. The qRT-PCR results showed that the expression of *LRP2*, *SLC39A14*, and *SLC39A8* increased by 69.10% (*p* < 0.05), 77.72% (*p* < 0.01), and 180.78% (*p* < 0.01), respectively, after 1 month of 50 mg/L Cd treatment compared with the control group (Figure 7A). Moreover, the expression of iron import-related genes *CUBN*, *LRP2*, *SLC39A14*, and *SLC39A8* decreased by 30.16% (*p* < 0.05), 40.86% (*p* < 0.01), 64.38% (*p* < 0.05), and 43.73% (*p* < 0.05), respectively, after 6 months of 50 mg/L Cd treatment (Figure 7B). However, the expression of divalent metal ion transporter *SLC11A2*, iron reduction-related gene *STEAP3*, and iron export-related gene *SLC40A1* was not significantly changed after 6 months of Cd treatment (*p* > 0.05) (Figure 7B). In addition, the in vitro results showed that *SLC11A2*, *CUBN*, *LRP2*, *SLC39A14*, *SLC39A8*, and *SLC40A1* gene expressions were significantly decreased after 5 or 10 μM Cd treatment (*p* < 0.05 or *p* < 0.01) (Figure 7C,D). These results suggested that Cd exposure could reduce the iron import capacity of rat kidneys and proximal tubular cells.

### 2.5. Cd-Induced Rat Proximal Tubular Cell Damage Was Alleviated by Fer-1 and NAC but Aggravated by Erastin and DFO

**Figure 8:**
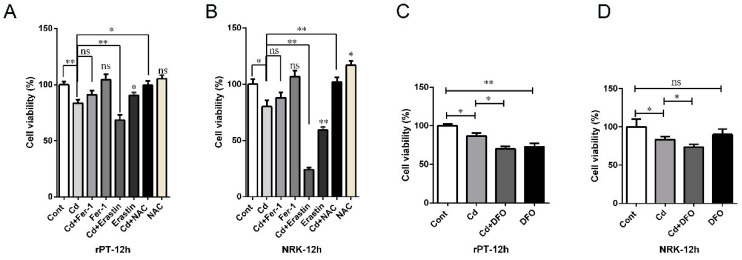
The effect of ferroptosis or iron homeostasis intervention on the cell viability of rat proximal tubular cells. NRK-52E and rPT cells were pretreated with ferroptosis inhibitor FER-1, ferroptosis inducer Erastin, powerful antioxidant NAC (**A**,**B**), and iron chelator DFO (**C**,**D**), and the cell survival rate was detected by CCK-8 assay. Each experiment was duplicated at least three times. (ns, not significant; * *p* < 0.05, ** *p* < 0.01).

Next, we further investigated the role of oxidative stress and iron metabolism disorders in Cd-induced kidney injury in vitro. The proximal tubular cells were pretreated with Ferrostatin-1 (Fer-1), a lipophilic ferroptosis inhibitor, Erastin, a ferroptosis inducer, and NAcetyl-L-cysteine (NAC), a reactive oxygen species scavenger, to investigate the role of this specific ferroptosis mode in Cd-induced kidney injury in vitro. The results showed that the cell viability of rPT cells was increased by 9.06% (*p* > 0.05) and 19.13% (*p* < 0.05) in Fer-1 + Cd-treated and NAC + Cd-treated groups, respectively, while it decreased by 17.99% (*p* < 0.01) in the Erastin + Cd group compared to the 5 μM Cd-treated group (Figure 8A). In addition, we also added deferoxamine mesylate (DFO), an iron chelator, to detect changes in cell viability. Notably, the cell viability of rPT cells was decreased by 19.27% (*p* < 0.05) in the DFO + Cd-treated group compared with the Cd-treated group, and the viability of cells in the DFO-alone treatment group was reduced by 27.24% (*p* < 0.01) compared to the control group (Figure 8C). In addition, the effect of Fer 1, NAC, Erastin, or DFO co-treatment with Cd on the reduction in NRK cell activity, induced by Cd, was consistent with that of rPT cells (Figure 8B,D). These results suggest that intervention with lipophilic antioxidants or reactive oxygen scavengers can partially rescue Cd-induced proximal tubular cell injury, but iron chelation can exacerbate cell injury.

## 3. Discussion

Chronic kidney disease has been recognized as a significant global public health problem, and there is a need to develop effective therapies to prevent its progression [32,33]. This study outlines the potential mechanisms of Cd exposure on kidney and proximal tubular cell injury. The results of our study showed that Cd exposure caused oxidative stress and chronic kidney injury in rats both in vivo and in vitro. Next, Cd exposure induced kidney iron deficiency by interfering with the iron metabolism both in vivo and in vitro; moreover, antioxidants and membrane lipid peroxide scavengers inhibited Cd-induced cell damage, further exacerbated by chelating iron and activating ferroptosis. In conclusion, Cd could induce kidney iron deficiency and chronic kidney injury in rats by interfering with the iron metabolism but not by inducing typical ferroptosis.

As an essential excretory organ of the body, the kidney is the main target organ for Cd accumulation. Studies have reported that Cd exposure can cause nephrotoxicity in mammals and birds, mainly reflected in renal structural destruction and dysfunction [34,35,36]. Consistent with previous studies, our results showed that Cd exposure caused structural abnormalities in tissues and mitochondrial ultrastructure damage, increased the MDA content, decreased the GSH content in rat kidneys, and increased the CREA and BUN contents in serum. It has been reported that renal tubules are the target of Cd-induced nephrotoxicity [12,37,38]. Meanwhile, the results of the in vitro experiments showed that Cd treatment caused morphological damage and decreased the cell viability of rPT cells, accompanied by the destruction of the mitochondrial ultrastructure, increased the ROS content, decreased the GSH content, and increased the MDA content. The results verified that Cd exposure could cause chronic kidney injury and proximal tubular cell damage by inducing oxidative stress [7,13,39,40].

Previous studies have shown that ferroptosis is associated with the biotoxicity of Cd in mammals [31,41,42]. Ferroptosis is characterized by damage to the antioxidant system, lipid peroxidation, and iron accumulation. Iron accumulation can drive membrane lipid peroxidation through the Fenton reaction, leading to changes in biofilms’ fluidity and permeability. It has been reported that Cd could increase the iron content, inhibit lipid peroxidation repair, and induce lipid peroxidation in Cd-induced acute kidney and liver injury [41,43]. Similarly, our results showed that the protein expression of GPX4 decreased and the protein expression of COX2 increased in Cd-induced chronic kidney injury in vivo and in vitro. However, our results showed that the total iron or divalent iron content decreased in Cd-induced chronic kidney injury and rPT cell injury. In addition, ACSL4 promotes ferroptosis by catalyzing the esterification of free fatty acids to form membrane phospholipids, and the inhibition of ACSL4 proteins significantly prevents ferroptosis [44]. Decreased FTH1 protein expression is involved in Cd-induced renal and hepatic ferroptosis by reducing iron storage and increasing iron release [41,43]. Another study found that Cd treatment reduced the expression of FTH1 proteins and induced ferroptosis in the swine cerebrum but increased FTH1 protein expression and inhibited ferroptosis in the cerebellum [31]. Our study found that Cd caused an increase in FTH1 protein expression in vivo and in vitro and a decrease in ACSL4 protein expression in vitro. We speculate that the two distinct effects of Cd on ferroptosis may be related to different organs and animal species, as well as the exposure time and dose. Our results indicated that Cd could not induce typical ferroptosis, as evidenced by the decreased or divalent iron content, increased FTH1 expression, and decreased ACSL4 expression in Cd-induced chronic kidney injury and proximal tubular cell damage.

Ferritinophagy (aka ferritin autophagy) is often associated with ferroptosis. Ferritinophagy is a selective autophagic process that relies on the receptor NCOA4 to degrade ferritin, especially FTH1, which can play a negative role in ferroptosis by binding iron to reduce free iron in cells [45,46]. Defective autophagy leads to intracellular iron consumption and reduced lipid peroxidation, leading to cell survival during Erastin-induced ferroptosis [47]. However, excessive autophagy can increase free iron in the cytoplasm by degrading ferritin and inducing TFRC expression [41,43,48]. Our results show that Cd can cause an increase in FTH1 protein expression in both in vivo and in vitro. In previous studies in our group, it has been demonstrated that Cd treatment can cause a blockage of the autophagic flow in kidney and rPT cells, involving inhibition of autophagosome and lysosome fusion and disruption of lysosome function [49,50,51]. It has been reported that iron dysregulation by endoplasmic reticulum stress (ERS)-mediated ferritinophagy contributed to Cd-induced ferroptosis in the mouse kidney [41]. Therefore, the increased expression of the FTH1 protein may be related to the block of autophagic flow and the increase in the gene transcription level caused by Cd. Our results indicated that Cd could increase FTH1 expression, which may be why Cd does not cause ferroptosis in the rat kidney.

Dysregulation of the iron metabolism is involved in heavy metal-induced biotoxicity. It has been reported that Cd treatment could cause an increase in iron content in the kidneys or testes [21,42,52,53]. Studies with contradictory results have reported that Cd reduces iron levels in the body, possibly involving a reduction in iron absorption by Cd through competitive binding to divalent metal cation transport receptors in the small intestine [19,54,55]. Previous studies have found that Cd, as a divalent metal cation, can interfere competitively with the absorption of other divalent metal ions, such as iron, copper, and manganese, through divalent metal cation channels in the digestive tract (especially in the small intestine), causing essential trace element deficiency diseases such as anemia [22,23]. The iron absorption mechanism is similar to that of other divalent metals, particularly lead and Cd, and dietary iron deficiency can further exacerbate absorption of the lead and Cd [17,56]. In the present study, the expression of the iron import-related gene SLC11A2 was increased, while the expression of the iron export-related gene SLC40A1 was decreased in the small intestine, and the HGB content and RBC count were slightly reduced, and more importantly, the iron content in serum and urine was also reduced after Cd exposure for 6 months. These results suggest that Cd causes a decrease in iron absorption in the duodenum and a deficiency in circulating iron. In addition, we investigated the effect of Cd exposure on the expression of iron transport-related proteins in rat kidney injury both in vivo and in vitro. The results showed that the expressions of iron export-related genes *CUBIN*, *LRP2*, *SLC39A14*, and *SLC39A8* were decreased in rat kidney and rPT cells, and the expression of the iron export-related gene SLC40A1 was decreased only in rPT cells after Cd exposure. It has been reported that Cd exposure can cause renal tubular damage and reabsorption dysfunction, which is a potential cause of Fanconi nephrotic syndrome [5,57]. We hypothesized that in addition to decreased iron absorption in the duodenal, renal, and tubular iron deficiency induced by Cd, it may be closely related to impaired tubular reabsorption. Our results also found that iron chelating agent DFO exacerbated the Cd-induced reduction in cell viability, suggesting that iron deficiency contributes to Cd-induced proximal tubule cell damage. Our results indicated that Cd could interfere with the circulating and renal iron metabolism, associated with iron deficiency and chronic kidney injury induced by Cd exposure.

## 4. Materials and Methods

### 4.1. Chemicals and Reagents

Cadmium chloride (CdCl_2_, 202908), NAC (30498), and DFO (D9533) were obtained from Sigma-Aldrich (Sigma, St. Louis, MO, USA). Dulbecco’s modified Eagle’s medium (DMEM, Gibco, 12800-017), fetal bovine serum (FBS, Gibco, 10437-028), Trizol (Ambion, 15596026), and trypsin-EDTA (25200072) were obtained from Thermo Fisher Scientific (Waltham, MA, USA). The ROS detection kit (S0033S), Mito-Tracker Red fluorescent probe (C1035), and Bicinchoninic acid (BCA) protein assay kit (P0012) were from Beyotime Biotechnology Co., Ltd. (Beyotime, Shanghai, China). The MDA (A003-1-2) and GSH detection kits (A006-2-1) were purchased from Nanjing Jiancheng Bio-Engineering Institute Co., Ltd. (Nanjing, China). The Cell Counting Kit-8 (CCK-8, A311-01) was purchased from Vazyme Biotechnology Co., Ltd. (Vazyme, Nanjing, China). The PrimeScrip^TM^ RT Reagent Kit (RR037A) and SYBR Green™ Premix Ex Taq™ (RR390A) were obtained from Takara Biomedical Technology Co., Ltd. (Takara, Beijing, China). The RIPA lysate (20101ES60) was obtained from New Cell & Molecule Biotechnology Co., Ltd. (NCM, Suzhou, China). The Fer-1 (HY-100579) and Erastin (HY-15763) were purchased from MedChemExpress LLC (MCE, Princeton, NJ, USA). All chemicals were of the highest purity grade available.

### 4.2. Animals and Treatments

All animal care and experimental procedures in this study were approved by the Animal Ethics Committee of Yangzhou University (approval ID: SYXK [Su] 2017-0044). Five-week-old Sprague Dawley (SD) male rats (160–180 g) were purchased from the Comparative Medicine Center of Yangzhou University. Different types of kidney injury models were established by drinking water with CdCl_2_ for different exposure times. (1) Establishment of subacute kidney injury mode: 5-week-old SD rats were randomly divided into three groups, with 6 rats in each group. The rats were exposed to CdCl_2_ by drinking water freely—control group (only purified water), Cd-treated group (purified water containing 50 or 75 mg/L CdCl_2_)—and were fed continuously for 1 month. (2) Establishment of chronic kidney injury model: 5-week-old SD rats were treated the same as the subacute kidney injury model and fed continuously for 6 months. The dose and exposure routes of CdCl_2_ used were referenced in previous studies [58,59]. The above animal experiments were performed without food or water 12 h before the end of the experiment. At the end of the experiment, the SD rats were sacrificed after anesthesia with an intraperitoneal injection of 2% sodium pentobarbital, and the renal tissues were separated and placed in neutral tissue fixative or transmission electron microscope fixative or stored at −80 °C for subsequent experiments.

### 4.3. Isolation and Culture of Renal Tubular Epithelial Cells

Isolation, identification, and culture of the rPT cells were performed according to previous descriptions [60]. Briefly, 21-day-old SD rats were anesthetized, sacrificed by cervical detachment, sterilized with 75% alcohol, and placed on the biosafety cabinet. Subsequently, the renal tissue fragments were obtained by kidney extraction, separation of the renal cortex, mechanical grinding, and centrifugation. The tissue precipitate was digested by collagenase IV in a 37 °C water bath for 15 min. The tissue was dispersed thoroughly by blowing with a dropper at the end of the process, and the proximal tubule segments were obtained after centrifugation. The tubular segments were resuspended and seeded in cell culture flasks or plates for subsequent experiments.

NRK-52E cells were subcultured according to the previous report [38]. First, the cells were washed thrice with PBS and were digested with 0.25% trypsin for 60 s. The digestion process was terminated by adding a complete culture medium with twice the volume of trypsin. Then, the cells were blown off with a dropper, transferred to a centrifuge tube, and centrifuged at 1000 rpm for 10 min. Cell precipitates were resuspended and seeded in cell culture plates for subsequent experiments.

### 4.4. Detection of Cell Viability by CCK-8 Assay

Cell viability measurement was performed as previously reported [38]. Briefly, the rat proximal tubule cells in the logarithmic growth phase in 96-well plates were treated with different concentrations of Cd for 12 h, and this was repeated six times within each group. In addition, blank control wells (with culture medium only) were set up. After Cd treatment, 10 μL of CCK-8 reagent was added to each well, which was shaken and mixed gently and then incubated in the cell incubator in the dark for 1–2 h. OD values were determined using a full-wavelength microplate reader (BioTek, Winooski, VT, USA) at a wavelength of 450 nm. The cell viability was calculated according to the formula [(OD treatment group-OD blank group/OD control-OD blank group)] × 100%.

### 4.5. Detection of Cadmium and Iron Contents

The contents of Cd and Fe were measured according to previous reports [61]. Renal tissue samples were placed in an oven for 48 h to ensure that the sample was dehydrated, and then nitric acid (excellent grade pure) was added and placed on a microwave digester for digestion according to the manufacturer’s instructions (Intertek, London, UK). At the end of digestion, the dissolved solution was transferred to a constant volume flask, and ultrapure water was added to a constant volume and mixed thoroughly upside down. In addition, samples of blood, serum, and urine were processed the same way as tissue samples, except that they did not need to be dried. The samples’ Cd or Fe content was analyzed using flame atomic absorption spectrophotometer (PerkinElmer, Waltham, MA, USA).

### 4.6. Detection of Blood Routine and Serum Biochemistry

The whole blood of rats was placed in an incubator at 37 °C for 15 min, and then serum was collected by centrifugation at 2500 rpm for 10 min. The CREA and BUN levels were measured using an AU5800 automatic biochemical analyzer (Beckman Coulter, Brea, CA, USA) to assess renal function. The determination of CREA and BUN content was performed as previously reported [36].

Routine blood test was performed according to the previous study [62]. The whole blood of rats was collected in a centrifuge tube containing anticoagulant, mixed upside down, and the HGB and the number of RBC in the blood were detected using automatic blood analyzer (Abacus, Budapest, Hungary).

### 4.7. Detection of MDA and GSH Contents

The contents of MDA and GSH were determined according to the previous study [63]. The protein concentration of the samples was determined by BCA protein assay kit to normalize the level.

### 4.8. Detection of ROS Content by Flow Cytometry

At the end of the Cd treatment, the cells were collected by 0.25% trypsin digestion and centrifugation. rPT cell precipitates were resuspended in PBS, filtered through a 200-mesh screen, and centrifuged again. DCFH-DA fluorescent probes were diluted in a 1:1000 ratio using a serum-free cell culture medium. The cell precipitates were resuspended in the above working solution, and then incubated at 37 °C in the dark for 20 min, shaking once every 3–5 min. At the end of incubation, ROS levels were measured using flow cytometry (BD, Franklin Lakes, NJ, USA) after washing with a serum-free cell culture medium. Measurement of intracellular ROS was performed as previously described [14].

### 4.9. Histopathological Observation

H&E staining and histological analysis were performed according to a previous study [61]. Briefly, the kidney tissues were dissected and immediately fixed in 10% formaldehyde buffer, after which tissues were embedded in paraffin. The slice thickness was adjusted to 5 micron sections and flattened on the slide. Renal tissue sections were stained with hematoxylin-eosin (H&E), and the tissue structure was observed under a light microscope (Leica, Wetzlar, Germany).

### 4.10. Observation of Mitochondrial Ultrastructure and Reticular Structure

rPT cells in the logarithmic growth phase were treated with different concentrations of Cd for 12 h. The Mito-Tracker Red fluorescent probe was diluted with cell culture medium to a final concentration of 75 nM. The above working solution was added to the cell culture plate, and then the cells were placed in an incubator at 37 °C in the dark for 30 min to load the probes. After incubation, the cells were washed with PBS and added to Hank’s Balanced Salt Solution (HBSS). Confocal fluorescence microscopy (Leica, Wetzlar, DEU) was used to observe the mitochondrial reticular structure. Immunofluorescence assays were referenced to previous studies [14].

The mitochondrial ultrastructural changes in the kidney issues and cells were observed by transmission electron microscopy (HT7700; Hitachi, Japan). The study followed the method mentioned earlier [36].

### 4.11. Real-Time Quantitative Polymerase Chain Reaction

Total RNA was isolated from tissue or cell samples using Trizol reagent. The steps for RNA reverse transcription and qRT-qPCR were the same as before [36]. Total RNA concentration and purity were determined using Nanodrop 2000 software (Thermo Scientific, Waltham, MA USA). The extracted mRNA was reverse-transcribed into cDNA using the PrimeScrip^TM^ RT Reagent Kit. Specific oligonucleotide primers for *TFRC*, *SLC11A2*, *SLC40A1*, *SLC7A11*, *GPX4, NRF2*, *IREB2*, *FTH1*, *HSBP1*, *ACSF2*, *ACSL4*, *LRP2*, *SLC39A14, SLC39A8*, *STEAP3*, and *β-ACTB* were designed based on sequences already deposited in GenBank using Primer 5.0 software (Premier, CA, USA). The primers for RT-qPCR analysis are shown in Table S1. qRT-PCR was performed using SYBR Green™ Premix Ex Taq™ (Takara, Japan). All responses were performed by a two-step method using the 7500 Real-Time PCR System (Applied Biosystems, Shanghai, China). Normalization of mRNA levels was achieved with *β-ACTB* levels and evaluation with the 2^−ΔΔCT^ method. Heat maps were drawn using the software Origin 2021 (Origin, Northampton, MA, USA).

### 4.12. Western Blot Analysis

Protein extraction and Western blot analysis were performed with reference to our previous study [64]. Total proteins of tissues and cells were extracted with RIPA lysate, and protein concentration was determined with a BCA protein assay kit. The extracted proteins were denatured by adding 5× SDS buffer in boiling water for 10 min. Protein samples were separated by SDS-PAGE and transferred to polyvinylidene difluoride (PVDF) membranes (Merck, Cork, Ireland) by wet transfer. After sealing the PVDF membrane with 5% nonfat milk, the PVDF membrane was incubated with diluted anti-SLC7A11 (A2413, ABclonal, Wuhan, China), anti-GPX4 (67763, Proteintech, Wuhan, China), anti-COX2 (12282, CST, Danvers, MA, USA), anti-ACSL4 (22401, Proteintech, Wuhan, China), anti-VDAC3 (ER1918-49, Huabio, Hangzhou, China), anti-Fth1 (4393S, CST, Danvers, MA, USA), and anti-β-actin (4970L, CST, Danvers, MA, USA) overnight at 4 °C. After incubation with the primary antibody and washing, the PVDF membranes were incubated with the anti-rabbit or anti-mouse secondary antibody (CST, Danvers, MA, USA) for approximately 2 h at room temperature and were washed with TBST. These protein bands were visualized by the Tanon chemiluminescence Imaging Analysis system (Tanon, Shanghai, China) using the ECL kit (NCM, Shanghai, China). β-actin was used to normalize protein expression levels. Optical density analysis was performed using Image J 1.42q software (NIH, Bethesda, MD, USA).

### 4.13. Statistical Analysis

The present results were collected from at least three independent experiments, and all the data were expressed as mean ± standard error of the mean (SEM). Homogenous data were analyzed by one-way analysis of variance (ANOVA) using IBM SPSS Statistics 19 statistical software (IBM, Armonk, NY, USA), and the post hoc analysis test was performed by using least-significant difference (LSD). The tested data agree with the normal distribution. *p* ≥ 0.05 means that there is no significant difference between different groups; *p* < 0.05 means that the difference between different groups is significant; *p* < 0.01 implies that the difference between different groups is extremely significant. GraphPad Prism 6 software (San Diego, CA, USA) was used to draw charts.

## 5. Conclusions

In summary, our study demonstrated that Cd exposure induced partial ferroptosis-related features in rat kidneys or proximal tubule cells, such as oxidative stress, lipid peroxide accumulation, and GSH/GPX4 axis inhibition, but it did not cause typical ferroptosis due to a decreased iron content, decreased ACSL4 protein expression, and increased FTH1 protein expression. Second, Cd caused decreased iron import in the duodenum and reduced iron absorption in the kidney and proximal tubule, leading to a decreased circulating iron content and renal iron deficiency. Finally, the ferroptosis inducer Erastin and iron chelator DFO aggravated Cd-induced proximal tubule cell damage but were alleviated by Fer-1 and NAC. Our results provide new insights into the nephrotoxicity that is induced by Cd exposure, which will help identify effective targets for preventing and treating Cd-induced diseases.

## Data Availability

The datasets used and/or analyzed during the current study are available from the corresponding author on reasonable request.

## Supplementary Materials

The following supporting information can be downloaded at https://www.mdpi.com/article/10.3390/ijms25020763/s1.
