# Supplementary material for: Cadmium Induces Kidney Iron Deficiency and Chronic Kidney Injury by Interfering with the Iron Metabolism in Rats
Source: Int J Mol Sci. 2024 Jan 7;25(2):763. doi: 10.3390/ijms25020763 (PMC10815742; doi:10.3390/ijms25020763)
Supplement: Supplementary file 1 [file ijms-25-00763-s001.zip › ijms-2787479-supplementary.pdf]

Table S1. Primer sequences of mRNA for qRT-PCR

Gene	Primer (5'→3')	Product length/bp	Genbank No
SLC40A1 F	TGCCAGACTTAAAGTGGCCC	188 bp	NC_051344.1
SLC40A1 R	CAGTACTGGCCAAATTCGCA		
ACSF2 F	CCAGTTACGATTTCACGACCA	195 bp	NC_051345.1
ACSF2 R	TGCCTACACTTCCAGCCTTCT		
IREB2 F	GAGACTGGGCTGCGAAAGGA	124 bp	NC_051343.1
IREB2 R	CCTGGGAGGAACTCAAGTGGTG		
FTH1 F	TTCAGGGCCACATCATCCCG	129 bp	NC_051336.1
FTH1 R	GCAAGTGCGCCAGAACTACC		
TFRC F	TGAAACTGGCTGCAGATGAG	221 bp	NC_051346.1
TFRC R	TTCTGACTTGTCCGCCTCTT		
SLC11A2 F	AGTGAAACCCAGCCAAAG	276 bp	NC_051342.1
SLC11A2 R	TCAGCAAAGACGGAGACG		
SLC7A11 F	GTTCAGACGATTGTCAGACAGAA	113 bp	NC_051337.1
SLC7A11 R	GGCAGATGGCCAAGGATTTG		
GPX4 F	AGTCCTAGGAAGCGCCCAG	178 bp	NC_051342.1
GPX4 R	CATCGCGGGATGCACACAAG		
ACSL4 F	TGGGCTGACAGAATCATGCG	130 bp	NC_051356.1
ACSL4 R	AACTGTATAACCACCTTCCTGC		
HSPB1 F	CGGCAACTCAGCAGCGGTGTCT	160 bp	NC_051347.1
HSPB1 R	CATGTTCATCCTGCCTTTCTTCGTG		
SLC39A8 F	TTTGTGGGGCTGGCTATTGG	197 bp	NC_051337.1
SLC39A8 R	TGAGTATGGTCATTCTGGCCG		
SLC39A14 F	ACTTACTTCATCGCCTTGTCCA	198 bp	NC_051350.1
SLC39A14 R	TCCGTGATGGTGCTCGTTTT		
STEAP3 F	GTCAACCTGGCTGTGAAGCA	199 bp	NC_051348.1
STEAP3 R	AGCCCAGCGTGGACTGC		

Gene	Primer (5’→3’)	Product length/bp	Genbank No
LRP2 F	GAGAAAAACTGCCCTCCCCA	178 bp	NC_051338.1
LRP2 R	AGAGGCACTGGAACTCGTTG		
CUBN F	TGAAGCTCAGCCTCCATTCAA	194 bp	NC_051352.1
CUBN R	ACCTCACGGTTAGGGCATTG		
NRF2 F	GTCAGCTACTCCCAGGTTGC	138 bp	NC_051338.1
NRF2 R	CAGGGCAAGCGACTGAAATG		
β-ACTB F	AACCTTCTTGCAGCTCCTCC	190 bp	NC_051347.1
β-ACTB R	TACCCACCATCACACCCTGG		
